# Talimogene laherparepvec (T-VEC) and Emerging Intralesional Immunotherapies for Metastatic Melanoma: A Review

**DOI:** 10.1007/s11912-024-01611-9

**Published:** 2024-11-27

**Authors:** Simran Kalsi, Amanda L. Galenkamp, Rohit Singh, Atulya Aman Khosla, Peter McGranaghan, Jessica Cintolo-Gonzalez

**Affiliations:** 1 Larner College of Medicine at the University of Vermont, 89 Beaumont Avenue, Burlington, VT 05401 USA; 2 Department of Medicine, Division of Hematology and Oncology, University of Vermont Medical Center, 111 Colchester Avenue, Burlington, VT 05401 USA; 3 Corewell Health William Beaumont University Hospital, Royal Oak, MI 48073 USA; 4 Baptist Health South Florida, Miami, FL 33146 USA; 5 Semmelweis University, Budapest, Hungary; 6 Department of Surgery, Division of Surgical Oncology, University of Vermont Medical Center, 111 Colchester Avenue, Burlington, VT 05401 USA

**Keywords:** Metastatic Melanoma, Intralesional Therapy, T-VEC

## Abstract

**Purpose of review:**

As the incidence of cutaneous melanoma continues to rise worldwide, its heterogeneous presentation proves challenging for managing and preventing relapse.

**Recent findings:**

While surgery remains a mainstay in staging and treatment of locoregional metastatic melanoma, intralesional therapies have emerged as a new tool to treat unresectable in-transit and nodal metastases and reduce the risk of relapse through immunomodulatory mechanisms.

**Summary:**

In this review, we will provide an overview of intralesional therapies for melanoma with a particular focus on talimogene laherparepvec (T-VEC) and its future uses. We then discuss the landscape of current and emerging intralesional therapies.

## Introduction

Cutaneous melanoma is the fifth leading cause of cancer, with an estimated 197,700 new cases reported in 2022 in the United States and an increasing worldwide incidence [1]. It has the potential to metastasize throughout the body, including to internal organs as well as sites in the skin, soft tissue, and lymph nodes. While early stage (I-IIA) melanoma has 5-year overall survival rates of greater than 90%, it worsens in more advanced disease [1, 2] Melanoma arises in melanocytes due to a combination of genetic predisposition (CDKN2A, CDK4) and acquired mutations (BRAF, MEK) from ultraviolet light-induced damage.

Surgical management plays a pivotal role in early staging and treatment via wide local excision and sentinel lymph node biopsy when indicated. While systemic therapy has had a rising role in management of more advanced stage disease in the either the adjuvant (Stage IIB-IV) [3–5], neoadjuvant [6–9], and treatment settings [10–13], there has still remained a need for additional modalities for locoregional control in certain clinical settings. Locoregional control can include surgical metastasectomy for palliation and/or disease control [14, 15], isolated limb infusion or perfusion for progressing disease of the extremities [16], as well as intralesional therapies [17]. While systemic therapy has become a preferred option to integrate into cases of locoregional metastases, there remains a role for intralesional therapies. [18]

Given the range of options available for clinical metastases to the skin, soft tissues, and lymph nodes, an understanding of the available intralesional agents and how and when to incorporate them into treatment regimens is continuing to evolve. Herein, we summarize the landscape of intralesional therapies for melanoma, first reviewing historic agents, followed by current and future applications of the only FDA-approved intralesional therapy, talimogene laherparepvec (T-VEC), and emerging therapies for future consideration. An overview of the therapies described in this review is provided in Table 1.

**Table 1:** Summary of current and investigational intralesional therapies for melanoma

Intralesional Therapy	Mechanism of Action	Indications	Adverse Effects
*FDA-Approved Agents*			
Talimogene laherparepvec (T-VEC)	Oncolytic virus (modified HSV-1) stimulating GM-CSF	Stage IIIB-IVM1a advanced unresectable cutaneous melanoma	Mild constitutional symptoms, local injection site reactions, vitiligo
*Emerging Investigational Agents*			
PV-10	10% Rose Bengal xanthene dye stimulating lysosomal release and tumor cell lysis	Stage IIIB-IVM1c advanced unresectable cutaneous melanoma	Transient local injection site pain, pruritis, local inflammation
SD-101	Synthetic CpG oligonucleotide stimulating CD8 + T cell via TLR-9	Stage IIIB-IVM1c advanced unresectable cutaneous melanoma	Transient flu-like illness, local injection site pain
Tilsotolimod	Synthetic TLR-9 agonist stimulating Th-1 immunity	Stage IIIB-IVM1c advanced unresectable cutaneous melanoma	Constitutional symptoms, elevated aminotransferases, hepatitis, colitis
CMP-001	Synthetic CpG-A rich encapsulated virus-like particles	Stage IIIB-D advanced unresectable cutaneous melanoma	Transient flu-like illness, local injection site reactions
NKTR-262	TLR-7/8 agonist stimulating Th-1 immunity	Stage IIIB-IVM1a advanced unresectable cutaneous melanoma	Transient flu-like illness, rash, fatigue, pruritis, nausea
CAVATAK	Selective tumor cell lysis via ICAM-1 and DAF	Stage IIIB-IV melanoma progressed on single prior anti-PD-1 therapy	Pruritis, diarrhea
Pexa-Vec	Oncolytic virus stimulating GM-CSF and β-galactosidase	Stage IIIB-C advanced cutaneous melanoma	Injection site reactions
HF-10	Oncolytic virus (spontaneously modified HSV-1) stimulating intratumoral CD8 + T cell infiltration	Stage IIIB-IVM1a advanced unresectable cutaneous melanoma	Transient flu-like symptoms, fatigue, local injection site reactions
PVS-RIPO	Recombinant type I polio vaccine stimulating IFN inflammation	Stage IIIB-IV advanced unresectable cutaneous melanoma	Local injection site reactions, pruritis
Plasmid IL-12	Cytokine DNA stimulating multiple immune cells	Stage IIIB-IVM1c advanced melanoma	Constitutional symptoms, local injection site reactions
Vusolimogene oderparepvec (RP1)	Oncolytic virus (modified HSV-1) stimulating GM-CSF	Stage IIIB-IV advanced unresectable cutaneous melanoma with disease progression on anti-PD-1	Constitutional symptoms

## Historic Agents

### Bacille Calmette-Guerin (BCG)

Intralesional Bacille Calmette-Guerin (IL-BCG) is a live attenuated strain of Mycobacterium bovis that has historically been used to trigger a nonspecific immune response against metastatic melanoma at the injection site and in distal lesions. However, in a phase III randomized controlled trial, IL-BCG showed no significant difference in disease-free survival (DFS) or overall survival (OS) between cohorts treated with IL-BCG and observation alone (5-year survival 67% versus 62%, p = 0.40) or when combined with an alkylating agent, dacarbazine (DFS p = 0.74, OS p = 0.81). Toxicity was mild to moderate, including punctate abscesses in most patients. However, 10–13% of participants experienced systemic reactions and regional adenopathy, and one patient acquired a life-threatening infection during the study. Therefore, interest in BCG waned as its risks outweighed the benefits. [19]

A novel BCG lysate-containing thermosensitive hydrogel may provide an alternative to the live mycobacteria by inducing a T-cell mediated proinflammatory tumor microenvironment targeting melanoma. Preclinical data from Kremenovic et al. shows that BCG lysate thermosensitive hydrogel promoted systemic immunity, reduced metastasis, and prolonged survival more effectively than live BCG when injected adjacent to the tumor in murine models [20]. Findings need to be translated into clinical trials to assess if BCG lysate hydrogel can overcome the current limitations of live BCG in humans.

### Interleukin-2 (IL-2)

Interleukin-2 (IL-2) is a pleotropic cytokine that binds to IL-2 receptors on T cells to alter the immune response by stimulating cytotoxic T cells and natural killer cells. It has been more broadly studied for its role as a systemic immunotherapy, demonstrating durable responses in a subset of patients, but its widespread use is limited by its toxicity [21–23]. Systemic dose-limiting adverse effects include hypotension, edema, arrhythmias, hematologic toxicities, and renal toxicities [23]. As an intralesional therapy, IL-2 has shown response rates varying from 25–96% with complete clinical response in up to 50% of patients in early studies [21]. In a 2021 observational cohort study, 65 patients with in-transit melanoma were treated with intralesional IL-2 and followed for a median of 27 months. DFS and OS for patients who achieved complete response were 65.5% and 69.0%, respectively, with a median time to recurrence of 10.5 months. [22] While intralesional use of IL-2 reduces its toxicity, clinical application is significantly limited by its cost, extensive dosing regimen (requiring a mean of 10 injections over 12 weeks to achieve complete response), and lack of benefit in treating uninjected sites [23, 24]. Ongoing studies are assessing the efficacy of IL-2 with other drugs, including topical chemotherapy and checkpoint inhibitors, to reduce the dose needed to treat. [25–27]

### Interferon gamma (IFNγ)

Interferon gamma (IFNγ) is another pleotropic cytokine that modulates T-cell differentiation and anti-tumor immunity and has also been used as a systemic immunotherapy, though now replaced by the more efficacious checkpoint inhibitors. It has been used intralesionally for melanoma in small-scale studies showing increased circulating T cells and minimal adverse effects. However, a significant lack of persistent antitumor immunogenicity makes it a less viable intralesional option. [28]

### Velimogene Aliplasmid (Allovectin-7)

In progressive melanoma, major histocompatibility complex class I (MHC-I) expression is frequently altered, allowing tumor cells to evade immune detection and increasing their oncogenicity [29]. Velimogene aliplasmid (allovectin-7) is an intralesional gene therapy agent studied for the treatment of melanoma. Allovectin-7 is a plasmid DNA encoding for HLA-B7, which upregulates MHC-I molecules and induces a proinflammatory response.

Allovectin-7 was initially studied in several small phase 1 trials, reporting promising results and minimal toxicities [30–32]. Three phase 2 studies were conducted in patients with metastatic melanoma. Study size varied from 52–127 patients with metastatic melanoma, and ORR varied from 9–18% with no severe toxicities reported [33–35]. Based on these results, two phase 3 trials were initiated. The first compared the combination of dacarbazine/allovectin-7 versus dacarbazine alone and did not find a significant difference in median time to progression (1.9 vs. 1.6 months) or survival (10.8 vs. 9.2 months) between the two groups [36]. The second compared allovectin-7 to the physician’s choice of chemotherapy (dacarbazine or temozolomide) and was terminated early with no difference in ORR or OS at greater than 24 weeks. [37] There are currently no ongoing or planned trials for allovectin-7.

## Talimogene Laherparepvec (T-VEC)

### T-VEC Monotherapy

Talimogene laherparepvec (T-VEC) is an intralesional oncolytic virus immunotherapy FDA-approved for the treatment of stage IIIB-IVM1a advanced unresectable cutaneous melanoma. T-VEC is a herpes-simplex-virus-type-1 (HSV-1)-based intralesional injection composed of live attenuated virus re-engineered to replicate within tumor cells selectively. Once inside tumor cells, the oncolytic virus stimulates an immune response via the expression of granulocyte–macrophage colony-stimulating factor (GM-CSF), resulting in anti-tumor responses at both the injected site and systemically [38]. Treatment can be continued until complete response, patient intolerance, or confirmed progressive disease.

In the pivotal OPTiM randomized open-label phase III trial, 436 patients with unresectable stage IIIB-IVM1c melanoma received treatment with either T-VEC or GM-CSF for six months. Participants were followed over three years to assess overall survival and complete response. In the final analysis, T-VEC had a median overall survival of 23.3 months (95% CI 19.5–29.6) compared to 18.9 months (95% CI 16–23.7) in those treated with GM-CSF. In addition, 16.9% of patients (N = 50) in the T-VEC arm achieved complete response compared to only 0.7% (N = 1) in the GM-CSF arm [39]. In a recent meta-analysis, the pooled complete- and overall response rates to T-VEC monotherapy were 30% and 44%, respectively (N = 642) [40]. In all studies, T-VEC was shown to have a tolerable safety profile. The most commonly reported side effects are mild constitutional symptoms and local injection site reactions [39–41]. However, as with other systemic immunotherapies, vitiligo has been reported to occur in some patients after treatment with T-VEC, likely due to an overactive anti-melanocyte immune response. [42]

For select patients who achieved an initial complete response (CR) with T-VEC monotherapy, re-treatment with T-VEC may be an option for recurrent melanoma. A small study of five patients at the Netherlands Cancer Institute assessed the response to re-introducing T-VEC monotherapy in recurrent melanoma and monitored for associated adverse effects. All patients initially received treatment with a median of eight T-VEC courses for in-transit melanoma and achieved CR. After melanoma recurrence, 60% of patients achieved histologic and/or imaging proven CR again after a median of three additional cycles of T-VEC. The remaining 40% required ongoing treatment with T-VEC at the time of study publication [43]. None of the patients in this study experienced distance metastases; the adverse effects were mild, including fatigue, flu-like symptoms, and injection site pain.

### Neoadjuvant T-VEC

Overall survival and recurrence-free survival for T-VEC as a neoadjuvant to surgery is currently being investigated. In an ongoing, randomized open-label phase II trial, 150 patients with advanced resectable stage IIIB-IVM1a melanoma received treatment with either T-VEC followed by surgery or surgery alone. After a two-year follow-up, overall survival and recurrence-free survival were 88.9% and 29.5% for those treated with neoadjuvant T-VEC versus 77.4% and 16.5% in those treated with surgery alone (overall survival 80% CI = 0.58–0.96, recurrence-free survival 80% CI = 0.30–0.79) [44]. Adverse events were consistent with the aforementioned studies, including mild constitutional symptoms and injection site reactions. These findings indicate an estimated 25% reduction in the risk of disease recurrence for neoadjuvant T-VEC compared with surgery alone with minimal burden of adverse effects.

T-VEC is also being investigated with or without radiotherapy as part of an open-label phase 2 trial at Memorial Sloan Kettering Cancer Center. Patients with melanoma, Merkel cell carcinoma, or other solid tumors with skin metastasis are randomized to either T-VEC with hypofractionated radiotherapy or T-VEC alone and will be assessed for partial or complete clinical response. [45]

While neoadjuvant systemic therapy is becoming commonly employed for resectable stage III and IV melanoma, integrating T-VEC with systemic therapy is also under investigation. The NIVEC trial is a single arm phase II open-label trial of patient who received neoadjuvant T-VEC plus nivolumab combination therapy for resectable early metastatic (stage IIB/C/D-IV M1a) melanoma. They report acceptable toxicity and a major pathologic response (complete plus near-complete response) rate of 65% [46]. Adding T-VEC to systemic immunotherapy may be a possible strategy to increase pathologic response with an acceptable toxicity profile as compared to dual systemic therapy options.

### T-VEC Combination Therapy

Given the role of mutations in BRAF and MEK in melanoma progression, BRAF/MEK inhibition has been investigated in combination with T-VEC to augment its activity in tumor cells. Preclinical data in murine models and human xenografts has found that T-VEC, combined with selective MEK 1/2 inhibitor trametinib, enhances oncolytic viral replication to enhance melanoma cell death and delay tumor growth. T-VEC and MEK inhibition together increased accumulation of cytotoxic T cells and expression of programmed cell death 1 (PD-1) in the tumor microenvironment [47]. Triple therapy with T-VEC, MEK inhibition, and an anti-PD-1 antibody was then found to further augment immune response with over 80% complete tumor eradication from baseline and increased overall survival without overt toxicity in pre-clinical murine models [47, 48]. A phase 1b clinical trial (NCT03088176) is currently recruiting participants to study T-VEC in combination with BRAF and MEK inhibitors, dabrafenib and trametinib, for the treatment of advanced BRAF-positive melanoma. [49]

Oncolytic viral therapies and immune checkpoint inhibitors (ICIs) should theoretically also have synergistic effects to overcome the limitations of resistance to ICI. To date, phase 1b-3 clinical trials have evaluated or are currently investigating the use of T-VEC in combination with ipilimumab, pembrolizumab, and nivolumab.

Ipilimumab is a CTLA-4 inhibitor that enhances T-cell recruitment and activation. In an open-label phase 1b trial, 18 patients with stage IIIB-IVM1c melanoma were treated with T-VEC and ipilimumab in an alternating regimen. Durable CR was observed in 22% of patients with an objective response rate of 50%. Incidences of adverse effects were consistent with known safety profiles for each agent, and no new toxicities were observed. [50]

A subsequent open-label phase 2 trial assessed the safety and efficacy of TVEC in combination with ipilimumab versus ipilimumab alone in patients with unresectable stage IIIB-IVM1c melanoma. 198 patients were randomized to receive T-VEC plus ipilimumab or ipilimumab alone. Results showed a significantly higher objective response rate of 39% in the combination therapy arm versus 18% in the ipilimumab arm (OR 2.9; 95% CI 1.5 to 5.5; P = 0.002). Additionally, 52% of patients in the combination arm observed decreases in visceral lesions versus 23% in the ipilimumab arm [50]. Adverse effects were largely consistent with known safety profiles for each agent with three patients in the combination arm experiencing non-treatment related fatal AEs. [51, 52]

Pembrolizumab binds to PD-1, blocking its receptor and ligands from binding. This increases the activity of antitumor T cells. In a global phase III trial, 692 patients with stage IIIb-IVM1c unresectable melanoma were randomly assigned to T-VEC-pembrolizumab or placebo-pembrolizumab. Progression-free survival (PFS), overall survival (OS), complete response rate (CRR), and safety were assessed. T-VEC-pembrolizumab did not significantly improve PFS (hazard ratio, 0.86; 95% CI 0.71 to 1.04; P = 0.13) or OS (hazard ratio, 0.96; 95% CI 0.76 to 1.22; P = 0.74) and did not significantly improve CRR when compared with placebo-pembrolizumab (17.9% in treatment arm vs. 11.6% in placebo arm) [53]. Incidences of adverse events in each arm were similar and consistent with known adverse effects for T-VEC and pembrolizumab. While these results call into the question the role of combination therapy in the first line setting, retrospective studies have supported the role of T-VEC as monotherapy or in combination with immunotherapy as a salvage regimen after progression or recurrence on systemic immunotherapy [54–56]. This combination is still actively under investigation for anti-PD-1 refractory melanoma. [57, 58]

A single arm open-label phase 2 trial at the Netherlands Cancer Institute is also actively investigating TVEC and ICI combination therapy in a neoadjuvant setting using the anti-PD-1 agent, nivolumab. Patients with stage IIIB-IVM1a melanoma are eligible to enroll for treatment with neoadjuvant T-VEC and nivolumab therapy. The primary endpoint is the pathologic complete response rate. Secondary endpoints are the rate of delays or failures to perform surgery, event-free survival, collection of tumor material for biomarker development, and safety. [59, 60]

While studies have shown that combining TVEC with ICIs may improve anti-tumor response, a recently published study from Leung et al. shows that the rate of cutaneous immune-related adverse events (cirAE) is increased twofold in TVEC plus ICI therapy versus ICI alone (hazard ratio: 2.03, P = 0.006) [61, 62]. Toxicities are dose-dependent and present heterogeneously ranging from pruritus to toxic epidermal necrolysis. [63] Management with topical or systemic corticosteroids is frequently used and, when studied after nivolumab therapy, use has been shown to have no negative impact on the rate or quality of antitumor responses [64]. However, careful collaboration between oncologists and dermatologists may be necessary for patients to continue their immunotherapy.

### Limitations and Future Directions of T-VEC

Based on the data summarized above, T-VEC may provide a greater benefit in durable control of unresectable locoregional melanoma rather than significantly improving overall survival. However, efficacy increases with combination therapy. The best regimen and timing for combination therapy with T-VEC remain under ongoing investigation, not only for melanoma but also for other soft tissue malignancies and solid tumors. Challenges in these studies include dose-dependent toxicities and safety concerns of combination therapy, though results appear promising.

Beyond expanding the use of T-VEC to treat other types of cancer, predictive biomarkers are an early area of investigation to assess for response to therapy. In the future, biomarkers may identify patients more likely to respond to treatment with T-VEC and its varying combination therapies. This would be of particular benefit to patients who are resistant to prior treatment with anti-PD-1 therapy. Biomarker development is a stated endpoint for the ongoing study from the Netherlands Cancer Institute on TVEC and nivolumab. [60]

Accessibility of T-VEC is another area of concern moving forward. In the clinical setting, storage, handling, and cost of T-VEC have proved challenging in implementing the therapy into standard practice. T-VEC requires storage at -80 °C, preparation in a sterile biosafety cabinet with differing doses for each cycle, and direct access to tumor lesions for manual administration. Many pharmacies do not have deep freezers or dedicated preparation spaces. Lesions may also regress or may not be measurable for dosing the intratumoral injection. Bedside ultrasound could be used as an aide in this process for lesions in the soft tissue or lymph nodes. Immunosuppression is generally a contraindication to T-VEC, given the increased risk for localized or disseminated herpes infection [65]. Biosafety concerns have also been raised, given that T-VEC uses a live replicating virus with a risk of transmission if there is accidental exposure (i.e., needlestick injury) during preparation or administration. However, T-VEC is sensitive to acyclovir for antiviral treatment should exposure occur. [64, 65] Therefore, there are existing guidelines for the safety and handling of this medication,63 and most institutions have specific standard operating procedures in place for the preparation, storage, and administration of T-VEC.

Finally, the financial cost of T-VEC and combination therapies with T-VEC is a significant limiting factor in its implementation. T-VEC therapy alone is estimated to cost approximately $362,033, escalating to over $490,000 when combined with ipilimumab [65]. Access to healthcare facilities and experienced providers able to offer T-VEC therapy can be limited and further exacerbated by the cost and frequency of dosing necessary to treat advanced melanoma.

## Other Investigational Agents

### PV-10 (10% Rose Bengal)

PV-10 is an intralesional therapy derived from Rose Bengal disodium. Rose Bengal is a water-soluble xanthene dye shown to demonstrate antitumor effects through a combination of lysosomal activity and T-cell activation. While some may think of this as a more of a historic agent, studies of PV-10 in conjunction with other therapies and its safety profile and fewer contraindications has led it to re-emerge as a potential intralesional therapy of interested.

A 2014 phase 2 multicenter clinical trial of single-agent intralesional PV-10 in patients with treatment-refractory melanoma demonstrated an overall response rate (ORR) of 51% and a complete response rate of 26% with a median time to response of 1.9 months. [66] Reported adverse events were mild, with the most common being injection site reactions. This study was continued in two single-center analyses for patients with in-transit melanoma, resulting in an overall response rate (ORR) of 52–87% and similar adverse events [67, 68]. Based on these results, a 2015 phase 3 randomized control trial sought to investigate PV-10 versus systemic chemotherapy or intralesional oncolytic viral therapy (dacarbazine, temozolomide, or TVEC) in patients with stage IIC/IIIB melanoma [69]. However, the study was terminated in 2019 due to inadequate enrollment.

Additional studies have evaluated the efficacy of PV-10 in combination with other antitumor agents. In 2017, a phase 2 open-label, non-comparative study analyzing the effects of intralesional PV-10 followed by radiotherapy in patients with in-transit disease (IIIB +) found an ORR of 87% [70]. The median time to peak response was 3.8 months, with an overall loco-regional progression rate of 80%. All 15 patients in this study experienced grade 1–2 adverse effects, most commonly injection site reactions, but were able to complete the treatment schedule. A randomized control trial investigating the efficacy of PV-10 in combination with ICIs is currently recruiting (NCT02557321) [71]. In phase Ib of the study, patients received a combination of PV-10 and pembrolizumab. Preliminary data showed an ORR of 65% in checkpoint-naïve patients, and an ORR of 29% in checkpoint-refractory patients [72, 73]. Phase II is ongoing with patients receiving either a combination of PV-10 and pembrolizumab or pembrolizumab alone.

PV-10 exhibits a unique antitumor effect, which has been proposed to be through lysosomal activation, excretion, and T-cell activation. Given this, its future use may best suit patients with advanced melanoma, melanoma resistant to traditional therapies, or patients with contra-indications to other therapies. Its efficacy and relatively low risk may make it an optimal choice as monotherapy or combined with other systemic treatments.

### Toll-like Receptor 9 Agonists (SD101, tilsotolimod, CMP001)

Toll-like receptors (TLRs) have been investigated as a potential treatment for several malignancies. TLR agonists improve tumor cell susceptibility to ICIs and induce dendritic cell maturation, cytokine excretion, antigen-presenting cell and natural killer cell activation, and T-cell responses [74]. These mechanisms help improve tumor cell susceptibility to ICIs.

SD-101 is a synthetic CpG oligonucleotide that stimulates TLR-9. A 2018 phase 1b study investigated if a combination of SD-101 and pembrolizumab in unresectable or metastatic melanoma patients could amplify the immune response enough at the primary injection site to affect distant lesions [75]. In anti-PD-1 naïve patients, the ORR was 78% with an estimated 12-month PFS rate of 88%. This decreased to a 12-month PFS of 15% in patients with a history of prior anti-PD-1 therapy. The most common side effects included injection site reactions and flu-like symptoms.

The phase 1/2 ILLUMINATE-204 trial studied a combination of TLR-9 agonist tilsotolimod and ipilimumab in patients with metastatic melanoma refractory to anti-PD-1 therapy [76]. Patients with combination therapy had an ORR of 38%. However, 48.4% of patients experienced grade 3 or 4 treatment emergent events (diarrhea, increased transaminases, colitis, fatigue; 4.8% each), and 32.3% experienced at least one serious adverse event. Phase 3 is ongoing.

A phase 1b trial investigated the combination therapy of TLR-9 agonist CMP-001 and pembrolizumab in patients with unresectable or metastatic melanoma with either stable disease after 12 weeks or progressive disease during or after anti-PD-1 therapy. In the first arm, lesions were injected with CMP-100 and pembrolizumab. In the second arm, patients received CMP-100 monotherapy. Results were promising, with a demonstrated ORR of 23% [77]. Currently, CMP-001 is being studies in the neoadjuvant setting in combination with PD-1 inhibition with results pending [78–80]. More information on efficacy and durability is needed for TLR-9 agonists to become a mainstay treatment for advanced melanoma.

### Toll-like Receptor 7/8 Agonists (NKTR-262)

NKTR-262 is a TLR-7/8 agonist that promotes the release of tumor antigens and increases CD8 + T cells and NK cells to exert antitumor effects. When used in combination with NKR-214 (bempehaldesleukin), it has been shown to induce inflammation in solid tumor microenvironments [81]. The REVEAL study completed phase 1 and 2 trials investigating the effects of NKTY-262 with bempehaldesleukin and nivolumab in patients with locally advanced and metastatic solid tumor malignancies, including melanoma. However, the study was recently terminated by the sponsor based on the inability of the treatment to improve PFS, ORR, or OS. [82]

### Other Oncolytic Viral Therapies (CAVATAK, Pexa-Vec, HF10, PVS-RIPO)

Advances in genetic engineering and viral genomics have allowed for the development of several additional OV therapies to treat melanoma. Herein, we discuss coxsackievirus A21 (CAVATAK), pexastimogene devacirepvec (Pexa-Vec), HF10, and PVSRIPO (Lerapolturev).

Coxsackievirus A21 (CVA21) has been shown to preferentially infect tumor cells via intercellular adhesion molecule 1 (ICAM-1) and decay-accelerating factor (DAF) [83]. Infection with CVA21 has been shown to selectively lyse tumor cells in vitro and in vivo [84]. The phase 1b MITCI study assessed the safety and efficacy of CAVATAK (CVA21) in combination with pembrolizumab in 50 adult patients with metastatic or unresectable stage IIIb/C or IV melanoma. Results of the study published in 2022 demonstrated an ORR of 30% in all treated patients (95% CI 18–45%). Of the patients who were anti-PDA-1 naïve, ORR was 47% (95% CI 23–72%). In patients with disease progression on prior anti-PDA-1 therapy, ORR was 21% (95% CI 9–39%). Tumor regression was seen in both injected and non-injected lesions with a median OS of 45.1 months (95% CI 28.3 months to not reached). The most commonly reported adverse effects were pruritis and diarrhea [85]. CAVATAK in combination with pembrolizumab is currently being evaluated in several clinical trials (NCT02565992, NCT04152863, NCT04303169). [86–88]

Pexa-Vec is an OV therapy genetically engineered to express human GM-CSF and β-galactosidase, resulting in antitumor activity similar to T-VEC therapy. Very few studies have investigated the toxicities and effectiveness of Pexa-Vec therapy in malignant melanoma. A small phase 1b study administered a single intravenous infusion of Pexa-Vec to 3 patients 14 days before scheduled surgical resection of stage IIIB/C melanoma. Results showed increased IFNγ expression associated with NK-cell activation and CD8 + T cell expansion within 24 h of infusion. In this study, 55% of patients (n = 5) experienced a grade 3 or 4 toxicity, including infected sarcoma, lymphopenia, neutropenia, and intraoperative hypotension [89]. An ongoing study investigating Pexa-Vec combined with a recombinant PD-L1 monoclonal antibody therapy in metastatic melanoma is currently recruiting (NCT04849260). [90]

HF10 is a mutant Herpes Simplex Virus type 1 (HSV-1) with oncolytic properties [91]. A 2017 phase II trial investigating the combination of intratumoral HF10 and ipilimumab in patients with unresectable metastatic melanoma demonstrated an ORR of 41% at 24 weeks. [92] A single-arm phase 2 trial investigated the safety and efficacy of 12 weeks of neoadjuvant nivolumab and HF10 in resectable stage IIB-IVMIa melanoma [93]. The study showed that 5 out of 6 participants had a CR (0% tumor viability) assessed via pathologic analysis of surgical specimens. However, the 6th patient had progressive disease preventing surgical resection, and the study was terminated in 2022 based on the data and safety monitoring committee (DSMC) recommendation.

PVSRIPO (Lerapolturev) is a recombinant, live-attenuated poliovirus Sabin type 1 carrying an internal ribosomal entry site (IRES) of human rhinovirus type 2 (HRV2). PVSRIPO preferentially enters melanoma cells due to a high CD155 poliovirus receptor expression rate [94]. A phase 1 trial investigated intralesional PVSRIPO in patients with unresectable melanoma and demonstrated an ORR of 33% [95]. A phase 2 trial is currently underway to compare PVSRIPO (Lerapolturev) as an intralesional monotherapy with a combination of PVSRIPO and anti-PD-1 therapy for unresectable anti-PD-1/L1 refractory stage III-IVM1b melanoma (NCT04577807). [96]

### Plasmid IL-12 (Tavokinogene Telseplasmid)

The cytokine interleukin 12 (IL-12) is triggered when pathogen-associated molecular pattern (PAMP) or danger-associated molecular pattern (DAMP) recognition stimulates the secretion of IFN- γ by T cells, NK cells, and dendritic cells. In a normal response, IL-12 reduces regulatory T cells and stimulates the expansion of dendritic cells. The exact mechanism of IL-12 is unknown, but a growing body of evidence suggests that IL-12 acts as an antitumor agent by regulating many types of immune cells including CD103 + dendritic cells and NK cells. [97]

Early studies have shown the efficacy of systemic IL-12 against advanced malignancies, albeit with significant toxicities [98, 99]. Because of these significant adverse effects, intralesional plasmid IL-12 was developed. Several clinical studies demonstrated that intratumoral injection of IL-12 (tavokinogene telseplasmid; “tavo”) leads to sustained local expression of IL-12 and can facilitate responses in both injected and uninjected tumors. This opened the door to intratumoral plasmid IL-12 therapy in patients with melanoma. [100, 101]

A 2020 phase 2 clinical trial examining the safety and efficacy of tavo followed by electroporation in patients with stage III/IV melanoma demonstrated an ORR of 35.7%, with 46% of all patients (n = 13) experiencing regression in uninjected lesions [101]. A separate phase 2 trial investigated the efficacy of tavo plus pembrolizumab in patients with metastatic or unresectable melanoma, showing an ORR of 41% and a CRR of 36% [102]. Neither study reported systemic symptoms classically associated with intravenous IL-12 therapy. Given the high response rate with a combination of tavo and anti-PD-1 therapy, tavo may be a useful addition to therapy in patients unlikely to respond to anti-PD-1 monotherapy.

### Vusolimogene oderparepvec (RP1)

With a similar mechanism to T-VEC, vusolimogene oderparepvec (RP1) is another recently developed intratumoral HSV-1-based oncolytic immunotherapy which expresses human GM-CSF to trigger anti-tumor activity. In promising phase 1/2 data from the IGNYTE study (NCT03767348), safety and efficacy of RP1 was assessed in patients with locally advanced or metastatic cutaneous melanoma that was confirmed to be progressive on an anti-PD-1 agent. Inclusion criteria included the presence of at least 1 measurable and injectable tumor with anti-PD-1 failure defined as confirmed disease progression after receiving the agent for at least 8 weeks. [103] In the 156 eligible and enrolled patients, RP1 was given at week 0 then every 2 weeks for a total of at least 8 cycles in combination with nivolumab beginning at week 2. After completing 8 cycles, patients transitioned to nivolumab monotherapy every 2 or 4 weeks for at least 2 years. Results showed an ORR of 31.4% with a CR in 12.2% of patients. Responses lasted at least 6 months from baseline with a median duration of response > 24 months. The most reported adverse events were grade 1–2 and included fatigue, fever, chills, and nausea. There was one reported case of immune-mediated myocarditis which was attributed to nivolumab [104]. A global phase 3 (IGNYTE-3) trial is currently underway with a target enrollment of 400 patients to evaluate OS, PFS, and ORR of RP1 plus nivolumab versus physician’s choice of treatment in patients with unresectable stage IIIb-IV cutaneous melanoma which has progressed on anti-PD-1 (NCT06264180). [105]

## Conclusion

In conclusion, the treatment landscape for cutaneous melanoma has seen substantial advances in recent years, and while there has been an emphasis and growth of systemic therapies, there remains a concurrent need for and growth of intralesional treatments. While traditional agents such as Bacille Calmette-Guerin (BCG), Interleukin-2 (IL-2), and Interferon-gamma (IFN) have shown promise in the past, their limits and side effects have prompted the search for more focused and effective alternatives. Some of the historic immunomodulating agents, such as Allovectin-7 or, more recently, Tavokinogene Telsplasmid, may warrant re-examination in conjunction with modern immunotherapies.

Talimogene Laherparepvec (T-VEC), an oncolytic viral immunotherapy approved by the FDA to treat advanced unresectable cutaneous melanoma, is one of the notable advances in intralesional therapy. T-VEC's effectiveness as a monotherapy and in conjunction with other therapies demonstrates its ability to improve patient outcomes. Its propensity to elicit full responses and its acceptable safety profile make it an attractive alternative for some individuals. Combination therapy utilizing T-VEC and immune checkpoint inhibitors (ICIs) have shown favorable results, but they are associated with an increased risk of cutaneous immune-related side effects. Notably, T-VEC has demonstrated an ability to elicit responses as salvage therapy even in the setting of disease progression on or after systemic immunotherapy and is being investigated in the neoadjuvant setting. Optimizing these combination therapies will require ongoing study and coordination among members of the multidisciplinary treatment team.

Despite all the advances, we face new obstacles regarding accessibility, affordability, toxicities and contraindications to certain therapies, and predictive biomarkers for these medicines. Biomarker research might assist in identifying individuals who are more likely to benefit from T-VEC and other treatments, allowing therapy to be tailored to individual requirements. Furthermore, overcoming logistical issues in drug storage, administration, and price load will be critical to ensuring greater access to intralesional therapies, such as T-VEC. The area of intralesional treatments for melanoma is quickly expanding, providing fresh hope for patients with advanced illness. While obstacles remain, continuing research and clinical trials offer the potential for more effective therapies and better results in the battle against cutaneous melanoma.

## Key References

- van Akkooi ACJ, Blank C, Eggermont AMM. Neo-adjuvant immunotherapy emerges as the best medical practice and will be the new standard of care for macroscopic stage III melanoma. Eur J Cancer. 2023;182:38-42. 10.1016/j.ejca.2023.01.004
  - Describes evidence from phase 1 and phase 2 trials that show the increased durable relapse free survival rates of neoadjuvant immunotherapy over adjuvant systemic therapy in stage III melanoma.
- Dummer R, Gyorki DE, Hyngstrom J, et al. Neoadjuvant talimogene laherparepvec plus surgery versus surgery alone for resectable stage IIIB-IVM1a melanoma: a randomized, open-label, phase 2 trial. Nat Med. 2021;27(10):1789-1796. 10.1038/s41591-021-01510-7
  - Describes data showing an improved recurrence-free survival rate for treatment with T-VEC plus surgery when compared to surgery alone in patients with stage IIB-IVM1a melanoma. This study highlights the potential long-term benefits of neoadjuvant intralesional therapy with T-VEC to treat advanced resectable melanoma.
- Kaufman HL, Shalhout SZ, Iodice G. Talimogene Laherparepvec: Moving From First-In-Class to Best-In-Class. Front Mol Biosci. 2022;9:834841. Published 2022 Feb 22. 10.3389/fmolb.2022.834841
  - Describes the bench-to-bedside process of bringing T-VEC to the appropriate melanoma patients and how biomarkers may be used to predict therapeutic response.

## Data Availability

No datasets were generated or analysed during the current study.
